# Preparation of *Gynostemma pentaphyllum* Extracts Using Natural Deep Eutectic Solvents with Ultrasound-Assisted Extraction for Cosmetic Applications

**DOI:** 10.3390/plants14111622

**Published:** 2025-05-26

**Authors:** Komcharn Jaikampan, Worrapan Poomanee, Thasang Thavanapong, Chuda Chittasupho, Kantamanee Jantadee, Mathukorn Sainakham

**Affiliations:** 1 Department of Pharmaceutical Sciences, Faculty of Pharmacy, Chiang Mai University, Chiang Mai 50200, Thailand; 2 Innovation Center for Holistic Health, Nutraceuticals, and Cosmeceuticals, Faculty of Pharmacy, Chiang Mai University, Chiang Mai 50200, Thailand

**Keywords:** *Gynostemma pentaphyllum*, deep eutectic solvent, ultrasound-assisted extraction, design of experiment, cosmetics

## Abstract

*Gynostemma pentaphyllum* (GP) is an herbal tea with medicinal properties and potent antioxidants. Deep eutectic solvents (DESs) are another interesting solvent for plant extraction due to their ability to extract plant phytochemicals efficiently. This research was conducted to study the phytochemicals of GP extracts isolated by DESs, investigate the biological activities, and develop cosmetic formulations containing GP extracts. The results showed that the total phenolic and total flavonoid contents of DES extracts were 0.39 ± 0.04 to 6.93 + 0.59 mg GAE/g extract and 1.48 ± 0.44 to 8.17 + 0.07 mg QE/g extract, respectively. The highest IC_50_ values of DES extract on DPPH assay, lipid peroxidation inhibition, and nitric oxide radical scavenging of DES extracts were 8.54 ± 3.31, 6.04 ± 0.82, and 38.63 ± 1.46 mg/mL, respectively. The DES extracts demonstrated collagenase enzyme inhibition at IC_50_ values of 0.92 ± 0.04 mg/mL. The selected DES extracts, S7, S9, S11, and S13, exhibited low cytotoxic effects on RAW264.7 cells and exhibited the most substantial reduction in nitic oxide levels. The selected DES extract with high bioactivities, S7, exhibited a high rutin and kaempferol content at 7.87 ± 0.01 mg rutin/g extract and 25.36 ± 0.08 mg kaempferol/g extract in the active content determination by HPLC assay. The cosmetic formulations containing S7 exhibited excellent stability after the stability test. This study illustrated the potential of DES extracts for further development in novel cosmetic products.

## 1. Introduction

Plants are widely used in many industries. Moreover, plants are a rich source of pharmacologically active compounds. They are used in various applications, including medicine, nutrition, perfumes, and cosmetics. Therefore, plants are an exciting source of raw materials; most have not yet been developed for potential use in cosmetics and pharmaceuticals. *Gynostemma pentaphyllum* (GP) (Jiaogulan) originates from China and is widely used in traditional Chinese medicine to treat several illnesses, such as coronary artery disease, diabetes mellitus, and liver disease [1,2]. Modern medical research has shown that GP has various pharmacological properties. Since GP has a wide range of biological activities, including anticancer, anti-inflammatory, neurologically protective, diabetic, antimetastatic, antioxidant, antifatigue, immune-regulating, and gut microflora-regulating properties due to its plenty of biological components, including saponins, polysaccharides, flavonoids, organic acids, trace elements, and a few other compounds, it is currently widely used in the development of pharmaceuticals and other medical care products to address the requirements of particular population groups [3]. Research has indicated that GP contains various chemical components, including phenolics and flavonoids [1,2,3], which act as good antioxidants, making it attractive to use GP extract to study and develop into cosmetic products.

Deep eutectic solvents (DESs) have accumulated interest as extraction solvents as an alternative to organic solvents. Considering DESs consist of natural components that act as hydrogen bond acceptors (HBAs) or hydrogen bond donors (HBDs), they have minimal toxicity, distinctive solvent qualities (incombustible, miscible in water, environmentally friendly, and harmless) [4], and an excellent possibility for extracting comparatively high levels of phytochemical compounds from natural sources for application in the pharmaceutical, cosmetic, and food industries. The features of DESs have much potential for use in extraction to identify natural compounds’ phytochemicals. In comparison to conventional solvents (for example, acetone, methanol, and ethanol), the majority of publishers have found that DES extraction provides high yields with the advantages of lower temperature, non-inflammable solvents, and a shorter duration of extraction [5,6]. Moreover, to extract scutellarin from *Erigerontis herba*, 27 DESs extracts were tested in conjunction with ultrasound-assisted extraction (UAE) in a previous investigation. The extraction yields of the 25 DESs extracted outperformed those of common solvents (methanol and 75% ethanol solution) [7]. Additionally, when compared to conventional solvent extracts, extracts containing DESs demonstrated superior antibiotic activity [8], antiproliferative activity of Caco-2 cancer cells and normal human keratinocytes [9], lower toxicity and improved accessibility of the specific components when delivered to mice [10], more potent antioxidants [11], and better anti-inflammatory properties including inhibitory activity of pancreatic alpha-glucosidase and lipase [12]. Because DESs extracts are bioactive, the pharmaceutical industry can employ them to heal and avoid illnesses or create new health care products.

In this study, GP was extracted utilizing a DESs to increase extraction yield and biological activity. The GP extracted with DESs and other conventional solvents was utilized to determine the phytochemical content, biological activities, and bioactive content via high-performance liquid chromatography (HPLC). The extract-containing emulsion formulations were optimized using the design of experiments (DoE) software version 10.0 (Stat-Ease, Minneapolis, MN, USA). Finally, the formulation properties and accelerated stability profile were assessed.

## 2. Results and Discussion

### 2.1. Extraction of GP Extracts by DESs

The result of deep eutectic solvent synthesis was shown in in Table 1. The solvent was a transparent liquid and light clear yellow liquid solution with a slight viscosity. In the preparation of the DESs, 20% water was added to reduce the solvent viscosity and enhance plant polyphenol extraction of the solvent.

### 2.2. Total Phenolic Content (TPC) and Total Flavonoid Content (TFC) Determination

The DES extracts from GP leaves and stems were measured for TPC. TPC of DES extracts ranged from 0.39 ± 0.04 to 6.93 + 0.59 mg GAE/g, as indicated in Table 1. The DES extract contains 88% lactic acid, and glucose from GP leaves (S7) showed the highest extraction yield of TPC in this study with a significant difference (*p* < 0.05) compared to other conventional solvents. In conventional solvent extracts, the extracts from water leaves (S1) gave the highest phenolic component extraction yield at 5.86 ± 0.15 mg GAE/g. The DES extracts from GP leaves and stems were also measured for TFC. In this study, TFC of DES extracts ranged from 1.48 ± 0.44 to 8.17 + 0.07 mg QE/g, as indicated in Table 1. The extract contains 88% lactic acid and glucose from GP leaves (S7) also showed the highest extraction yield of TFC with no significant difference with 95% ethanol leaves extract (S3) which yielded the highest TFC at 7.95 ± 0.83 mg QE/g. Previous studies indicated that DESs exhibited higher or comparable extraction efficiencies for TPC and TFC than conventional solvents. Attributed to the unique physicochemical properties of DESs, such as their enhanced solubility power of polar and non-polar compounds. Established on the properties of DESs, they were primarily hydrophilic but could also be more hydrophobic or hydrophilic. Because of its hydrophobic and hydrophilic characteristics, DES was compatible with both polar and non-polar plant components, increasing the effectiveness of extraction [8]. In order to extract phytochemical compounds from blueberry leaves, Santos-Martín et al. used a different DES made of lactate, sodium acetate, and water, and another made of choline chloride and oxalic acid. The results showed a preference for DES in extraction. The researchers found that DES recovered more phenolic compounds than conventional solvents, and the lactic-based DES allowed for the extraction of a diverse variety of amino acids and flavonol derivatives [13]. Previous research had demonstrated that adding water to DES could change its physical-chemical characteristics [14]. Adding water to DES could lower its viscosity through improved extraction efficiency and increased mass transfer from the solid material to the solution. However, it also made the DES more polar, which made it more suited to extracting more polar substances like flavonoids and phenolic compounds [15]. The specific combination and molar ratio of HBDs and HBAs significantly influenced the DES’s polarity, viscosity, and ability to solubilize different compounds. Different DES compositions had varying affinities for extracting phenolic compounds and flavonoids from the source material. For instance, more polar DESs might be more effective in extracting polar phenolic acids, while less polar DESs could be better for certain flavonoids [16].

### 2.3. In Vitro Antioxidant Activities Investigation of DESs GP Extracts

After determining the TPC and TFC, all GP extracts were determined for biological activity, including DPPH assay, lipid peroxidation assay, and metal chelating activity, as shown in Table 2. The DES extract with the highest DPPH radical scavenging was the leaf extract containing lactic acid and glucose (S7) with an IC_50_ value of 8.54 ± 3.31 mg/mL. The DES extract with the highest lipid peroxidation inhibition with an IC_50_ value of 6.04 ± 0.82 mg/mL was the leaf extract containing citric acid and fructose (S13). The highest inhibition of metal chelation at 60.97 ± 2.70% was obtained from the water extract (S1) at 500 mg/mL. In comparison, the DES extract with the highest inhibition of the metal chelation of a percentage inhibition value at 29.23 ± 1.24% was the leaf extract containing lactic acid and fructose (S9). As reported by these results, these extracts might exhibit potent antioxidant activity due to their high phytochemical content. Previous research demonstrated that using DES with lactic acid as a component, as opposed to 80% methanol, for the extraction of *Mentha pulegium* demonstrated the high amount of phytochemical compounds that resulted in potent antioxidant activity. It was found that DESs were great alternatives to other conventional solvents for the extraction of phenolic compounds to enhance antioxidant activity [17]. Additionally, the results of this study showed that DES outperformed conventional solvents due to its higher extraction yield and potential as an eco-friendly substitute solvent for improved free radical scavenging activity. However, EDTA was more effective than the DES extract with metal chelating activity in this study. From the results of the biological activity tests as previously described, the selected extracts S1, S2, S7, S8, S9, S10, S11, S12, S13, S14, and S15 were subjected to the next investigation of biological activities assay, namely, nitric oxide radical scavenging assay, FRAP assay, ABTS scavenging ability assay, and collagenase enzyme inhibition assay, as shown in Table 3.

In the nitric oxide radical scavenging assay, the deep eutectic solvent extract with the most effective NO radical scavenging activity was the lactic acid and glucose composition of the leaf extract (S7), with an IC_50_ value of 38.63 ± 1.46 mg/mL, which was higher than the water extract. The extract with the highest value of FRAP antioxidant activity had a FRAP value of 251.02 ± 11.38 mM Fe^2+^/g sample, which was the extract from water extraction (S1). The deep eutectic solvent with the highest FRAP value was the lactic acid and glucose (S7) extract, which had a FRAP value of 139.24 ± 2.83 mM Fe^2+^/g sample. The highest percentage inhibition of the ABTS scavenging ability test at 24.91 ± 2.47% was obtained from the solvent extract containing lactic acid and fructose (S9) at a concentration of 500 mg/mL, which was higher than the water extract but lower than ascorbic acid. According to previous studies, the DES-lactic acid-based extraction of *Curcuma aromatica* rhizomes inhibited NO radical scavenging by 48.86 ± 4.30% compared to 95% ethanol extract, with no significant difference at *p* < 0.05. Therefore, the NO radical scavenging inhibition was comparable to other conventional solvents [4]. From the collagenase enzyme inhibition test, the deep eutectic solvent extract with the highest collagenase inhibitory value was the solvent containing citric acid and fructose (S13) of the leaf extract with an IC_50_ value of 0.92 ± 0.04 mg/mL. In previous studies, two distinct concentrations of DES were used to study collagenase inhibition. Inhibition of 90% was demonstrated by the DES utilizing ChCl/glycerol and ChCl/urea at IC_50_ of 4.59 × 103 and 3.09 × 103 μM, respectively. Collagenase inhibition results were comparable to those of the aqueous solution, indicating that the chemical reaction of choline chloride and the hydrogen donor (glycerol or urea) exists to inhibit the collagenase enzyme [18,19]. Therefore, DESs showed promise in enhancing collagenase enzyme inhibition assay. Choline chloride-based DES demonstrated significant inhibition values of collagenase, indicating their potential as effective inhibitors in various cosmetic applications. Subsequently, the extracts S1, S7, S9, S11, and S13 were selected based on the previously mentioned bioactivity test results to examine the anti-inflammatory assay in macrophages.

Different DESs, with their unique combinations of hydrogen bond donors (HBDs) and acceptors (HBAs), possessed varying polarities, viscosities, and hydrogen bonding capabilities. These properties dictated their ability to solubilize and extract diverse antioxidant compounds from the source material. A DES better suited to dissolve and stabilize specific classes of antioxidants in the matrix will naturally yield an extract with higher activity. Some DESs might have a stronger affinity for extracting certain types of antioxidants (e.g., specific phenolic acids or flavonoids) over others. If the extracted compounds were potent antioxidants, the resulting extract would show higher activity in the assays. DESs might sometimes enhance the solubility and stability of specific bioactive compounds compared to traditional organic solvents or water, resulting in higher concentrations of active antioxidants in the extract [8].

### 2.4. Anti-Inflammatory Assay

#### 2.4.1. Cytotoxicity of DESs GP Extracts Against RAW264.7 Cells

**Figure 1:**
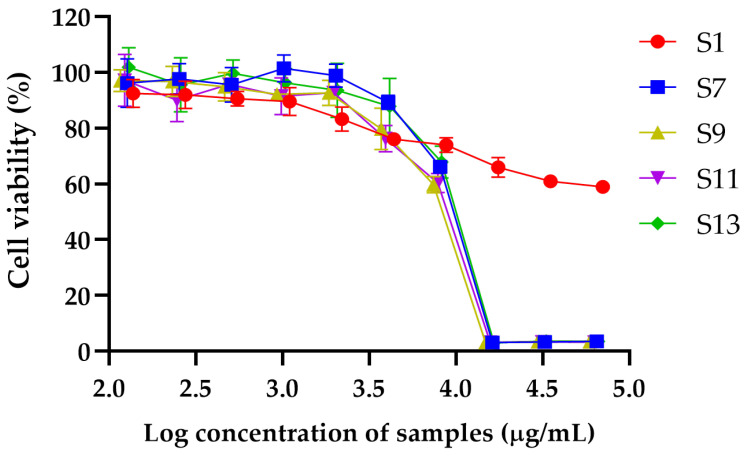
Dose-dependent cytotoxic effects of DESs extracts on cell viability. Graph illustrates percentage of cell viability following treatment with increasing log concentrations of S1, S7, S9, S11, and S13. Data are expressed as mean ± standard deviation (SD) of three independent experiments.

The cytotoxic effects of extracts S1, S7, S9, S11, and S13 were evaluated using the MTT assay, and IC_50_ values were calculated to determine the concentration required to inhibit 50% of RAW264.7 cell viability (Table 4). In addition, the maximum concentrations of each extract that maintained cell viability at or above 80% were identified as indicative of non-toxic levels (Figure 1). Among the tested samples, S1 exhibited the highest IC_50_ value (127.81 ± 41.43 mg/mL), indicating the lowest cytotoxicity and a favorable safety profile, thereby supporting its potential application in cosmetic formulations. It maintained 83.26 ± 4.30% cell viability at 2.20 mg/mL. S7 also showed a high IC_50_ value of 9.20 ± 0.15 mg/mL and preserved 89.20 ± 2.56% viability at 4.06 mg/mL, indicating low cytotoxicity. S11 demonstrated an IC_50_ of 8.28 ± 0.45 µg/mL, with cell viability of 92.74 ± 1.70% at 1.97 mg/mL, suggesting a similarly acceptable safety profile. S13 exhibited moderate cytotoxicity, with an IC_50_ of 8.80 ± 1.48 µg/mL. It maintained cell viability of 87.84 ± 9.99% up to 4.14 mg/mL. In contrast, S9 showed the lowest IC_50_ value (8.00 ± 0.19 mg/mL), indicating the highest cytotoxic potential among the tested samples. It maintained a cell viability of 79.77 ± 7.42% at 3.71 mg/mL, slightly below the 80% threshold for non-toxicity, reflecting a narrower safety margin.

#### 2.4.2. Effects of DESs GP Extracts on Nitric Oxide Secretion

**Figure 2:**
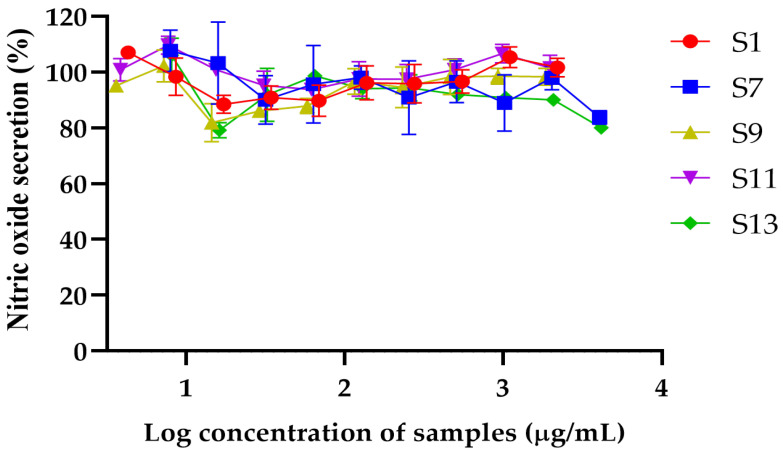
Percentage of nitric oxide secretion in response to varying log concentrations of S1, S7, S9, S11, and S13. Data are presented as mean ± standard deviation (SD) from three independent experiments.

The nitric oxide (NO) secretion levels were assessed for S1, S7, S9, S11, and S13 across various concentrations, revealing differences in their ability to modulate NO production (Figure 2). S13 exhibited the most substantial reduction in NO levels, achieving an 80.00 ± 1.44% secretion at the highest concentration of 4.14 mg/mL, indicating significant potential for anti-inflammatory activity. S7 demonstrated a consistent reduction in NO secretion, achieving a decrease to 83.73 ± 2.45% at the maximum concentration of 4.06 mg/mL, suggesting moderate anti-inflammatory potential. In contrast, S1, at the highest tested concentration of 2.2 mg/mL increased NO secretion, with NO levels recorded at 107.1% compared to LPS-treated control cells, indicating minimal impact on NO modulation. Similarly, S9 exhibited minor variations in NO secretion, with levels ranging from 95.2% at 3.61 mg/mL to 98.5% at 1.85 mg/mL, indicating limited activity in modulating NO levels. S11 showed relatively stable NO levels across concentrations, starting at 109.7% at 3.84 mg/mL and gradually declining to 93.8% at 61.56 µg/mL, stabilizing around 100.8% at higher concentrations, suggesting a weak effect on NO production. These results suggest that S1 and S7 were the safest samples with the broadest ranges of non-toxic concentrations, while S13 and S7 exhibited the most substantial reduction in NO levels, making them promising candidates for further exploration in cosmetic or anti-inflammatory formulations. Therefore, the extracts S1, S7, and S13 were selected based on the anti-inflammatory assay test results in macrophages to further examine the amount of active compounds using HPLC.

### 2.5. Bioactive Content Determination by HPLC Assay

**Figure 3:**
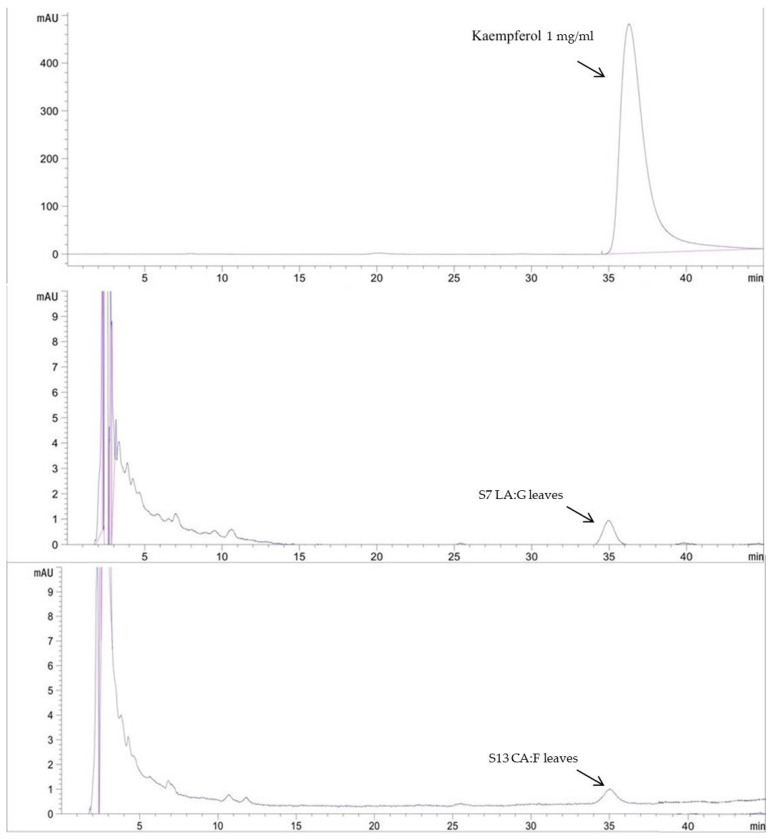
HPLC chromatogram of kaempferol standard and kaempferol content in selected DESs extracts.

**Figure 4:**
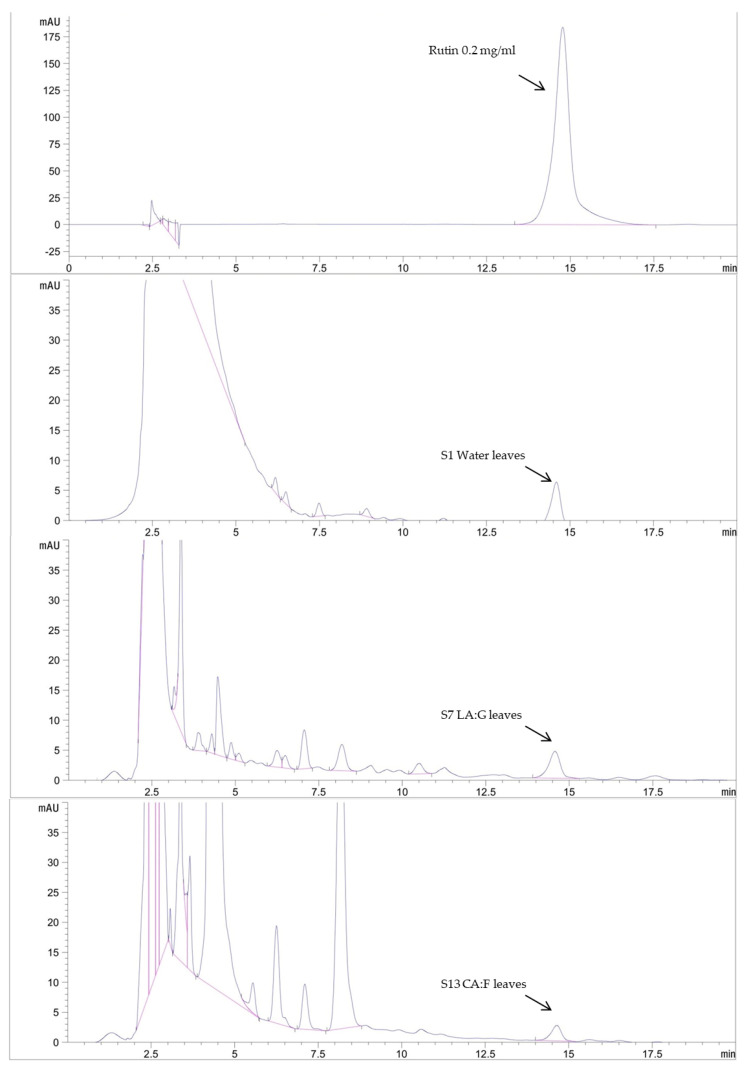
HPLC chromatogram of rutin standard and rutin content in selected DESs extracts.

HPLC analysis was used to analyze the active constituents of the extracts as shown in Table 5 and Figure 3 and Figure 4. Rutin and kaempferol were used as the standard for analysis; the water extract (S1) contained the highest amount of rutin at 8.95 ± 0.03 mg/g. The deep eutectic solvent with the highest rutin was the extract composed of lactic acid and glucose (S7) at 7.87 ± 0.01 mg/g. The extract with the highest amount of kaempferol at 25.36 ± 0.08 mg/g was the extract composed of lactic acid and glucose (S7). DESs could extract more active components than water solvents. According to a previous study, the HPLC analysis results show that the DES-lactic acid-based extraction of *Curcuma aromatica* rhizomes exhibited the high efficiency extraction of curcumin at 74.05 ± 0.86 µg/g [4]. Previous research by Xie et al. [20] also demonstrated the rutin content of GP with methanol as a conventional solvent. The results showed that the rutin content ranged from 0.01 to 21.49 mg/g. Therefore, the DESs serve as an alternative choice for obtaining safe and high-bioactivity extracts in this study for further cosmetic development.

For the bioactivities of selected extracts, S1, S7, and S13, preliminary phytochemical analysis was conducted using total flavonoid content to estimate the levels of bioactive compounds in the extracts. Based on these results, HPLC analysis was subsequently employed to identify suitable marker compounds that could represent the active constituents of the extracts. Among the identified compounds, rutin and kaempferol were consistently detected and therefore selected as representative markers. These markers were used as criteria for selecting extracts to be further developed into final cosmetic formulations. Interestingly, the selected deep eutectic solvent (DES) extracts, S7 and S13, exhibited rutin levels comparable to those found in the water extract (S1). Notably, kaempferol was detected at significantly higher concentrations in S7 and S13, while it was undetectable in S1. Previous studies had highlighted the importance of evaluating the bioactivities and chemical constituents of GP. For free radical scavenging activity, GP samples were evaluated by the DPPH radical scavenging activity. Extracts prepared using 50% acetone consistently exhibited high radical scavenging activity values, indicating efficient extraction of antioxidant compounds. Among the tested samples, the one crude extract exhibited the highest DPPH scavenging activity, reaching 402 µmol Trolox equivalents/g. Rutin and kaempferol, two major flavonoid constituents, were detected in varying concentrations across the samples. These findings suggest that rutin and kaempferol contribute significantly to the antioxidant potential of GP via their DPPH radical scavenging capacity [21]. In the lipid peroxidation inhibition study, rutin and kaempferol, the active compounds contained in GP, effectively inhibited lipid peroxidation with notable potency reflected in their IC_50_ values. Rutin, which contained a catechol group in the B-ring, exhibited strong antioxidant activity with an IC_50_ of approximately 10 µM, while kaempferol showed significant inhibition with an IC_50_ of approximately 23.8 µM, due to the presence of a 3-OH group supported by electron-donating substituents [22]. GP also exhibited anti-inflammatory activity in LPS-stimulated RAW264.7 cells by significantly reducing nitric oxide (NO) production in a dose-dependent manner. At 300 µg/mL, GP reduced NO levels by approximately 20% compared to the control. This effect was accompanied by the downregulation of NOS2 expression at both mRNA and protein levels, suggesting that GP inhibited inflammation via suppression of NO synthesis pathways. Rutin, identified as one of the major constituents of GP, exhibited strong anti-inflammatory activity by significantly reducing the levels of pro-inflammatory cytokines [23]. Moreover, kaempferol had also been shown to significantly inhibit inducible nitric oxide synthase (iNOS) expression at both mRNA and protein levels in cytokine-stimulated Chang Liver cells in a concentration-dependent manner. This leaded to reduce nitric oxide production, contributing to its anti-inflammatory effect [24].

### 2.6. Preparation of Cosmetic Formulations Contained DESs GP Extracts

#### 2.6.1. Formulation Optimization Using Design of Experiment (DOE)

**Figure 5:**
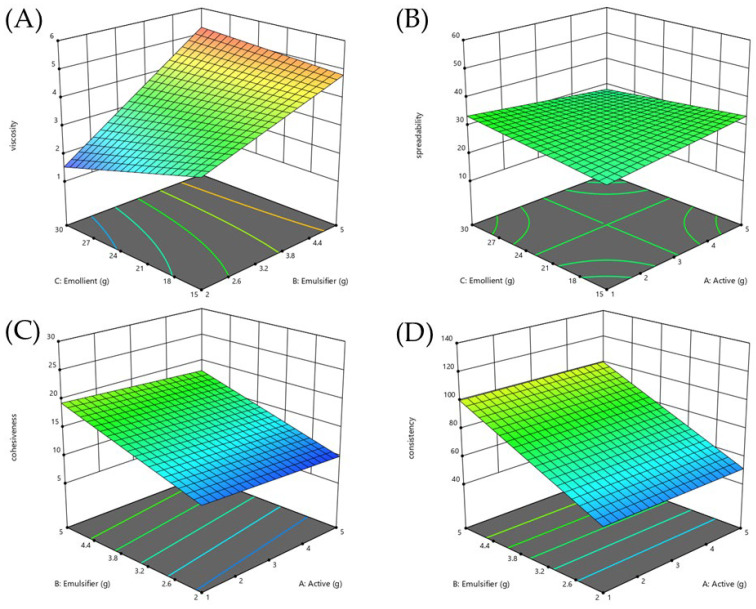
Response surface diagrams (RSD) illustrating interaction effects of independent variables influencing formulation viscosity (**A**), spreadability (**B**), cohesiveness (**C**), and consistency (**D**).

The description of eight formulations generated according to the 2^3^ full factorial design and response variables value from factorial design were shown in Table 6. The regression analysis and model performance summarized in Table 7 confirmed the reliability of all response models, with statistical significance (*p* < 0.05), R^2^ values greater than 0.8, differences between adjusted and predicted R^2^ within 0.2, and adequate precision values above 4. These models were subsequently used to interpret the variables’ effects and interactions on the measured responses. The control and optimized formulations following DES extract incorporation were compared as shown in Table 7. This study aimed to develop an emulsion formulation containing DES extract that was as similar to the control formulation as possible. The optimal condition was not the formulation with the best response value but the one with the response value most similar to the control formulation. After analyzing using DOE, F3 was the selected formulation, resulting in the optimal response surface diagrams with the most similar to the control formulation as shown in Figure 5. The optimized formulation (F3) was selected based on minimizing the percentage relative standard error (%RSE) across key physical properties, with a final ratio of DES extract:emulsifier:emollient at 1:2:15. Changes in formulation composition highlighted in Table 8 were made to compensate for the effects of DES extract addition while maintaining the physical attributes of the base formulation. The DES extract (β_A_) exhibited a slight positive but non-significant effect on viscosity. The emulsifier (β_B_) significantly increased viscosity, while the emollient (β_C_) significantly reduced it, as displayed in Figure 5A. To maintain viscosity close to the control, both emulsifier and emollient levels were reduced, resulting in a %RSE of 4.74%. Regarding spreadability, the addition of DES extract had no significant direct effect. However, the interaction between DES extract and emollient (β_AC_) showed a negative trend, suggesting that when combined, DES extract and emollient slightly reduced spreadability, although this effect was not statistically significant as shown in Figure 5B. Despite the reduction of the emulsifier, spreadability increased in the optimized formulation, yielding %RSE of 10.28%, slightly exceeding the acceptable threshold. The DES extract exhibited a slight non-significant negative effect regarding cohesiveness. The emulsifier and emollient both significantly increased cohesiveness, with a significant positive interaction (β_BC_) as displayed in Figure 5C. However, the three-way interaction involving DES extract, emulsifier, and emollient (β_ABC_) had a significant negative effect, indicating that the presence of the DES extract could weaken the combined positive contribution of emulsifier and emollient. Therefore, careful adjustment of emulsifier and emollient levels was necessary, and the resulting %RSE of cohesiveness was maintained at 1.40%. For consistency, the DES extract showed no direct significant effect, but the interaction between the DES extract and the emollient indicated a non-significant negative trend. The emulsifier significantly increased consistency as displayed in Figure 5D. After reducing the emulsifier content, a slight increase in consistency was observed, with a %RSE of 6.65%, which was still acceptable. Overall, the results suggest that although the DES extract alone had limited direct effects, its interaction with the emollient could influence spreadability, cohesiveness, and consistency. By reducing emulsifier and emollient levels while introducing DES extract at 1%, the optimized formulation (F3) successfully maintained key physical properties similar to the base formulation, particularly for viscosity and cohesiveness. Thus, F3 was selected for further stability evaluation.

#### 2.6.2. Accelerated Stability Test

**Figure 6:**
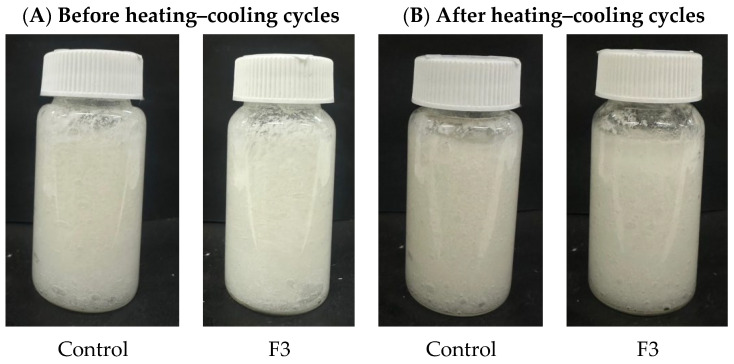
Physical appearances of control and F3 formulation before (**A**) and after (**B**) heating–cooling cycles test.

Accelerated stability tests are beneficial because they provide criteria for the physical stability of emulsions in a relatively short time. However, only studies under real storage conditions provide definitive results. The accelerated stability test was conducted to evaluate the stability of the control formulation and F3 under a heating–cooling cycle and centrifugation. This test aims to predict the product’s shelf life and assess any potential changes in its physical and chemical properties. The physical appearance of the formulation is displayed in Figure 6, and the formulation characterization after the heating–cooling cycle, including consistency, cohesiveness, viscosity, and spreadability, was shown in Table 9. The formulation containing DES extract demonstrated acceptable stability under accelerated conditions. The results showed no changes observed; color and texture remained stable and homogeneous, and there was no change in scent after the heating–cooling test. However, after the centrifugation test, the formulation shows the separation of the oil and water phases. Separation of cosmetic formulations after centrifugation can occur due to differences in density between the phases, leading to the lighter phase rising to the top. Factors such as emulsion instability and inadequate emulsifier concentration can also contribute to this phenomenon [25]. In the heating–cooling cycle test, the changes in the formulation characterization were observed. The response variables of both the control and formulation 3 (F3) decreased following the heating–cooling stability test. However, the stability of the formulations was not impacted. For instance, no phase separation or alteration in color and odor was observed after the stability test. Changes in the properties of cosmetic formulations after stability testing could be attributed to several factors, including chemical interactions among ingredients and temperature fluctuations. Additionally, improper storage conditions and the degradation of active ingredients could alter the formulation’s texture, color, and overall efficacy [26]. In this investigation, there were slight minor changes in consistency, cohesiveness, viscosity, and spreadability. After stability evaluation, the formulation preserved its integrity under varying temperature conditions by heating–cooling cycle test. The emulsion characteristics demonstrated satisfactory stability throughout the testing period, suggesting favorable physical stability. These results demonstrated that the formulation containing DES extract exhibited acceptable stability and could be applied to cosmetic formulations.

## 3. Materials and Methods

### 3.1. Chemical Materials

Folin-Ciocâlteu reagent, L-ascorbic acid, quercetin, gallic acid, rutin, kaempferol, 2,2- diphenyl-1-picryhydrazyl radical (DPPH), linoleic acid, dimethyl sulfoxide (DMSO), ferrous chloride (FeCl_2_), ethylenediaminetetraacetic acid (EDTA), sulfanilamide, sodium nitroprusside, N-(1-Naphthyl) ethylenediamine dihydrochloride (NED), 2,4,6-Tris(2-pyridyl)-s-triazine (TPTZ), ferrous sulfate (FeSO_4_), potassium persulfate (K_2_S_2_O_8_), 2,2′-azino-bis (3-ethylbenzthiazoline-6-sulphonic acid) (ABTS), calcium chloride (CaCl_2_), collagenase enzyme, 3,4-dihydroxyphenylacetaldehyde (DHPAA), and sodium periodate (NaIO_4_) were obtained from Sigma Chemicals Co., St. Louis, MO, USA.

### 3.2. Plant Materials

The dried leaves and stems of the plant were obtained from Lampang, Thailand. The dried leaves and stems were ground into a fine powder and kept in a well-closed container protected from light until further use.

### 3.3. Preparation of DESs

The DESs were prepared as previous study and indicated in Table 10 [4,27]. The preparation of DESs from various components and summarized the ratio that could form the appropriate solvents. Briefly, the components of HBAs and HBDs were combined in precise molar ratios and agitated at 80 °C to form a transparent liquid. This research selected these DESs, which are readily available and harmless for use in this study. DESs were kept at room temperature in sealed glass containers before usage. In order to enhance polyphenol diffusion and decrease viscosity, 80% (*v*/*v*) of all DESs were combined with 20% water. Furthermore, this study compared extraction with water and ethanol (50% and 95%).

### 3.4. Extraction

DESs, water, and ethanol solutions were performed to extract the leaves and stems of GP. A volume of 20 mL of ethanol solution (50% and 95%), water, and DESs were added to each sample. Each sample was subjected to 20 min of sonication in an ultrasonic cleaner. Following a 20 min centrifugation at 10,000 rpm, the extract was gathered and stored at 4 °C. Every extract was utilized for every analysis after being diluted 1:1 with DI water in the solution.

### 3.5. Preliminary Phytochemicals Analysis

#### 3.5.1. TPC Determination by Folin-Ciocâlteu Method

A 96-well test plate’s total phenolic content was determined using the Folin-Ciocâlteu test [4]. Volumes of 170 µL of 10% *v/v* Folin-Ciocâlteu reagent, 30 µL of 7.5% Na_2_CO_3_, and 10 µL of GP extract solution were added in a 96-well test plate. At room temperature, the plates were left in the dark for 30 min. The absorbance in milligrams of gallic acid equivalent per gram of extract (mg GAE/g extract) was measured using a microplate reader. The absorbance and gallic acid concentrations ranged from 0.16 to 200 µg/mL and were plotted against the standard curve for total phenolic content calculation.

#### 3.5.2. TFC Determination by Aluminum Chloride Colorimetric Method

The TFC was measured using the aluminum chloride colorimetric procedure [4]. In a 96-well plate, 30 µL of GP extract was mixed with 120 µL of distilled water, followed by the addition of 10 µL of 5% NaNO_2_. The mixture was given 5 min to incubate. A volume of 10 µL of 10% AlCl_3_ was added, and the mixture was then incubated for an additional 6 min. Then, 10 µL of distilled water and 60 µL of 1 M NaOH were added to the mixture. After 10 min of incubation, the absorbance was measured in a microplate and calculated as mg of quercetin equivalent per 1 g of extract (mg of quercetin equivalent QE/g of extract). The absorbance and quercetin concentrations ranged from 1.25 to 20 mg/mL and were plotted against the standard curve for total flavonoid content. The total flavonoid content of the extracts was determined in milligrams of quercetin equivalents (QE) per gram of extract.

### 3.6. In Vitro Biological Activities Determination

#### 3.6.1. Free Radical Scavenging Assay

Free radical scavenging activity was evaluated using the DPPH assay [28], which involved mixing 50 µL of GP extract with 50 µL of DPPH solution and letting it cover at room temperature for 30 min in the dark. A microplate reader was used to measure the absorbance and assess the DPPH free radical scavenging activity. The percentage of inhibition of free radical scavenging activity was computed using the following formula: [(Acontrol − Asample)/Acontrol] × 100 was used to compute the percentage of scavenging activity, where Acontrol and Asample represent the absorbance values of the control and sample groups, respectively. The IC_50_ value was the sample concentration that produced 50% DPPH scavenging.

#### 3.6.2. Lipid Peroxidation Inhibition Assay

Lipid peroxidation activity was measured using the ferric thiocyanate method [4]. Volumes of 50 µL of GP extract, 0.39 mg/mL ammonium thiocyanate, 50 µL ferrous chloride in HCl, and 50% (*v*/*v*) linoleic acid in DMSO were all included in the combination. The absorbance of the ferric thiocyanate complex at 490 nm was then measured to evaluate the extract’s inhibitory effect on lipid peroxidation. The percentage of lipid peroxidation inhibitory activity was then calculated using the following formula: % lipid peroxidation inhibitory activity = [(Acontrol − Asample)/Acontrol] × 100. The sample concentration that prevented 50% of lipid peroxidation is known as the IC_50_ value.

#### 3.6.3. Metal Chelating Activity

The metal chelating activity was investigated using the ferrous metal chelating method [29]. A volume of 100 µL of GP extracts was mixed with 50 µL of 2 mM FeCl_2_ solution and 50 µL of 5 mM ferrozine solution. The absorbance at 570 nm was measured after an incubation period of 15 min. Using EDTA as a reference, the percentage inhibition of ferrocene-Fe^2+^ complex formation was calculated using the following formula: The formula [(A − B)/A] × 100 was used to determine the percentage of metal chelating activity, where A means the control absorbance and B means the sample absorbance.

#### 3.6.4. Nitric Oxide (NO) Radical Scavenging Assay

Volumes of 20 µL of GP extract, 80 µL of 0.3% sodium nitroprusside, and 20 µL of PBS (pH 7.4) were combined on a 96-well plate for the NO radical scavenging experiment. Following 150 min of incubation, 60 µL of 0.1% N-(1-naphthyl) ethylenediamine dihydrochloride (NED) and 60 µL of 1% sulfanilamide in 2% H_3_PO_4_ were added to the mixture. The absorbance at 546 nm was measured after 10 and 5 min of incubation. The proportion of NO radical scavenging activity inhibition was computed using the formula: % NO inhibition = [(ABcontrol − ABsample)/ABcontrol] × 100 [4].

#### 3.6.5. Ferric Reducing Antioxidant Power (FRAP) Assay

The ferric reducing antioxidant power (FRAP) assay evaluated the extracts’ reducing capacity. The components of the FRAP test solution were 300 mM acetate buffer, 20 mM FeCl_3_^−^6H_2_O, and 10 mM TPTZ solution. After combining 180 µL of the FRAP test solution with 20 µL of the GP extract, it was left to sit in the dark for 30 min. Ferrous sulfate (FeSO_4_) served as the reference standard and ascorbic acid was used as a positive control. The absorbance at 593 nm for each concentration was then measured using a microplate reader. By building the regression of the absorbance against the concentration of FeSO_4_, the FRAP value of each sample was calculated, meaning that 1 mg of the sample is equivalent to 1 mg of FeSO_4_ [30].

#### 3.6.6. ABTS Scavenging Ability

An amount of 140 mM potassium persulfate (K_2_S_2_O_8_) was mixed with 7 mM ABTS to form an ABTS radical scavenging solution left overnight. Then, DI water was used to dilute the reactive ABTS for the experiment. A volume of 247 µL of ABTS solution was incubated with 20 µL of GP extract for 6 min. The absorbance was then measured at 734 nm using a microplate reader. The equation calculates the percent ABTS scavenging ability as [(ABcontrol − ABsample)/ABcontrol] × 100 = % NO inhibition [31].

#### 3.6.7. Collagenase Enzyme Inhibition

The collagenase enzyme solution, 10 mM calcium chloride (CaCl_2_), 125 mM borate buffer pH 7.5, and 50 µL GP extract were combined. The collagen solution and fluorescent reagent containing 0.75 mM DHPAA and 1.25 mM sodium periodate (NaIO_4_) were added after the combination had been incubated for 10 min at 37 °C in the dark and then for 60 min. The absorbance of the mixed solution was assessed by tracking the fluorescence reaction at 375 nm and 465 nm emission using a microplate reader (SpectraMax M3, Molecular Device, San Jose, CA, USA). The percentage of inhibition of collagenase was calculated. The IC_50_ value, the sample concentration that inhibited the collagenase enzyme by 50%, can be used to calculate the sample concentration that produced a 50% inhibition [31].

### 3.7. In Vitro Anti-Inflammatory Assay

#### 3.7.1. Cell Viability Assessment Using MTT Assay

The vitality of RAW 264.7 cells following treatment with the selected DES extracts was assessed using the MTT test. In order to allow for cell attachment, RAW 264.7 cells (8 × 10^3^ cells/well) were planted in 96-well culture plates and incubated for 24 h. After incubation, the cells were treated with the samples for 24 h in a medium devoid of serum, while the control group consisted of untreated cells. Following treatment, each well received 0.5 mg/mL of MTT solution after the medium was sucked. The cells were subsequently treated for 2 h to enable the development of formazan crystals. After removing the media, 100 µL of DMSO was added to each well to dissolve the formazan crystals and lyse the cells. To determine cell viability, absorbance was measured at 550 nm using a microplate reader (SpectraMax M3, Molecular Devices, San Jose, CA, USA) [32].

#### 3.7.2. Measurement of NO Production

The Griess assay was used to measure the production of NO from RAW 264. After being planted onto 96-well culture plates, 7 cells (8 × 10^3^ cells/well) were incubated for 24 h. After 3 h of treating cells with the selected DES extracts in a serum-free medium, the cells were stimulated with lipopolysaccharide (LPS; 5 µg/mL) for 24 h. The supernatants were cautiously moved to a fresh 96-well plate following the treatment time. After adding sulfanilamide and N-(1-naphthyl)ethylenediamine (NED) solutions to the supernatants, the plate was left in the dark for 10 min to allow for the colorimetric reaction, which was used to measure the NO levels. A microplate reader (SpectraMax M3, Molecular Devices, San Jose, CA, USA) was used to detect absorbance at 550 nm. The concentrations of nitrite were ascertained by comparing the absorbance values to a standard curve made from sodium nitrite. A 2-fold serial dilution was used to dilute the sodium nitrite standard, which was made at 300 µM [33].

### 3.8. Bioactive Content Determination by HPLC Assay

An HPLC system equipped with a UV-VIS detector (1260 Infinity II, Agilent Technologies, Santa Clara, CA, USA) and a 250 × 4.6 mm, 5 μm Capcell Pak C18 column (Shiseido Co., Ltd., Tokyo, Japan) was used to determine the amounts of rutin and kaempferol in the extracts. With an injection volume of 20 µL, a flow rate of 1.0 mL/min, and an absorbance of 254 nm, the mobile phase was composed of 15:85 (*v*/*v*) acetonitrile and 0.3% phosphoric acid in water [34]. The determination of kaempferol content requires the following parameters: an injection volume of 20 µL, an absorbance of 360 nm, a 47:53 (*v*/*v*) ratio of methanol to 0.4% phosphoric acid in water as the mobile phase, and a flow rate of 1.0 mL/min [35]. The rutin and kaempferol contents of the DES extracts were determined by comparing the HPLC chromatogram with a standard curve.

### 3.9. Preparation of Cosmetic Formulations

#### 3.9.1. Formulation Optimization Using DoE

A full two-level, three-factors (2^3^) factorial experimental design was carried out. The components used in the cosmetic emulsions are described and modified in Table 11 [36]. The amount of emulsifier, caprylic/capric triglycerides and DESs extract were selected as independent variables. Table 12 summarizes the experimental runs and the factor combinations used in this study. Eight formulations were prepared due to the combination of the factors (independent variables). The consistency, cohesiveness, viscosity, and spreadability were considered as the response variables (dependent variables) in this experiment. Eight different formulations were obtained from Design Expert 11.0.7.0 Software; Trial version (Informer Technologies, Inc., Los Angeles, CA, USA, 2020), as shown in Table 6, in which variable codes and actual values of each factor were demonstrated. After the experiments were done, the data were statistically analyzed by ANOVA with a level of significance less than 0.05. The response surface diagram and its mathematical equation for each response were generated as the following equation:

Predicted value = β_0_ + β_a_A + β_b_B + β_c_C + β_ab_AB + β_ac_AC + β_bc_BC + β_abc_ABC

The control formulation and the optimized formulations were compared by the percentage of relative standard error (% RSE) of the responses. The suitable model was indicated by an equation where the % RSE is less than 5%. This study aimed to develop an emulsion formulation containing DES extract that was as close to the control formulation as possible, with the response variables not significantly different from the control formulation. The optimal condition was the one with the response variables closest to the control formulation.

$$\%\mathrm{RSE}=\left|\frac{\mathrm{Predicted}\;\mathrm{Value}-\mathrm{Actual}\;\mathrm{Value}}{\mathrm{Predicted}\;\mathrm{Value}}\right|\times 100$$

#### 3.9.2. Preparation of Cosmetic Formulations Contain DES GP Extract

The emulsion formulations were prepared in a 250 mL beaker. The DES extract (S7) was chosen for the emulsion preparation. The oil and water phases were prepared independently. Xanthan gum was dissolved in glycerol, then added to the aqueous phase containing water, DES extract, and phenoxyethanol. The mixture was agitated at 80 °C until homogeneity was observed. The oil phase contains caprylic/capric triglycerides and hydrogenated lecithin, which were homogenized by stirring at 80 °C. The oil phase was gradually incorporated into the water phase while stirring for 10 min [37].

#### 3.9.3. Formulations Characterization

The formulation characterization, including consistency, cohesiveness, viscosity, and spreadability, were observed using a texture analyzer (TA-XT Plus C; Stable Micro Systems, Godalming, UK). The texture analyzer conditions were used according to the previous study by Tai et al. [38]. The viscosity property of the formulations was measured by a rheometer (R/S, AMETEK Brookfield, Middleborough, MA, USA) with a plate and P25 plate at 25 °C using control shear rate mode (CSR). Shear rate measurements were performed for 60 s by variation from 0.1 to 150 s^−1^.

#### 3.9.4. Accelerated Stability Test

The accelerated stability of the formulations was investigated by a heating–cooling cycle and centrifugation test. The emulsion formulations were kept in a refrigerator (4 °C) changing to the oven (45 °C), every 24 h for six cycles [39]. The centrifugation test was performed at 3500 rpm for 20 min (Centrifuge MPW352R, MPW Med instrument, Warsaw, Poland) [40]. The characteristic changes were observed as previously described.

### 3.10. Statistical Analysis

The experimental results were presented as the mean ± standard deviation and analyzed for differences using SPSS statistics software version 17.0, using one-way ANOVA tests. The *p* < 0.05 level was considered statistically significant.

## 4. Conclusions

As demonstrated by this study, the results indicated that DES extract contained lactic acid and glucose (S7) provided the greatest TPC and TFC, which were higher than other solvents in the GP leaves extracts. S7 exhibited significant potency in the DPPH assay and a nitric oxide radical scavenging activity. In addition, S13 showed high potency of lipid peroxidation inhibition activity and demonstrated collagenase enzyme inhibition. The potent DES extracts were selected for investigation in cell culture and HPLC analysis. The selected DES extracts exhibited anti-inflammatory activity in RAW 264.7 cells without cytotoxicity. In the HPLC assay, S7 showed high rutin and kaempferol content at 7.87 ± 0.01 mg/g extract and 25.36 ± 0.08 mg/g extract, respectively, and was selected for formulation development. Following the heating–cooling cycle test, the formulation incorporating S7 showed excellent stability. These results demonstrate that S7 could be considered a new ingredient with high bioactivities for cosmetic applications.

## Figures and Tables

**Table 1:** Code of extracts, total phenolic content (TPC), and total flavonoid content (TFC) determination of DESs extracts from GP.

Code	Components	The Extraction Yield	
		TPC (mg GAE/g)	TFC (mg QE/g)
S1	Water Leaves	5.86 ± 0.15	6.70 ± 0.66
S2	Water Stem	4.73 ± 0.41	7.46 ± 0.13
S3	95% EtOH Leaves	0.59 ± 0.04	7.95 ± 0.83
S4	95% EtOH Stem	0.35 ± 0.04	3.41 ± 0.29
S5	50% EtOH Leaves	4.50 ± 0.33	5.63 ± 1.82
S6	50% EtOH Stem	4.12 ± 0.28	6.17 ± 0.46
S7	LA:G Leaves	6.93 ± 0.59 *	8.17 ± 0.07
S8	LA:G Stem	2.46 ± 0.16	1.89 ± 0.19
S9	LA:F Leaves	5.82 ± 0.56	5.08 ± 0.94
S10	LA:F Stem	3.16 ± 0.45	1.48 ± 0.44
S11	CA:G Leaves	3.05 ± 0.38	2.33 ± 0.11
S12	CA:G Stem	0.42 ± 0.21	1.75 ± 0.12
S13	CA:F Leaves	6.55 ± 0.10	2.44 ± 0.02
S14	CA:F Stem	3.54 ± 0.07	1.60 ± 0.48
S15	CA:S Leaves	3.51 ± 0.22	2.09 ± 0.93
S16	CA:S stem	0.62 ± 0.10	2.19 ± 0.29
S17	MA:G Leaves	1.38 ± 0.51	1.62 ± 0.64
S18	MA:G Stem	0.39 ± 0.04	1.60 ± 0.27
S19	MA:F Leaves	4.09 ± 0.62	2.63 ± 0.58
S20	MA:F Stem	2.45 ± 0.23	1.98 ± 0.23
S21	MA:S Leaves	4.43 ± 0.19	3.13 ± 0.28
S22	MA:S Stem	1.09 ± 0.09	2.88 ± 0.52

* = significantly higher difference in total phenolic content at *p* < 0.05 compared to conventional extracts (S1, S3 and S5).

**Table 2:** IC_50_ values (mg/mL) of free radical scavenging, lipid peroxidation inhibition, nitric oxide radical scavenging, and collagenase enzyme inhibition of DESs extracts.

Code	Components	Free Radical Scavenging	Lipid Peroxidation Inhibition	Nitric Oxide Radical Scavenging	Collagenase Enzyme Inhibition
S1	Water Leaves	30.28 ± 9.49	132.29 ± 74.89	-	-
S2	Water Stem	76.79 ± 29.03	>1000	>1000	-
S3	95% EtOH Leaves	>1000	>1000	-	-
S4	95% EtOH Stem	>1000	>1000	-	-
S5	50% EtOH Leaves	>1000	>1000	-	-
S6	50% EtOH Stem	644.76 ± 762.35	>1000	-	-
S7	LA:G Leaves	8.54 ± 3.31 *	8.38 ± 0.05 *	38.63 ± 1.46	1.27 ± 0.01
S8	LA:G Stem	55.71 ± 10.04	16.60 ± 7.40	74.60 ± 19.16	4.40 ± 0.07
S9	LA:F Leaves	9.91 ± 2.28 *	9.27 ± 2.58 *	67.20 ± 11.39	3.77 ± 0.52
S10	LA:F Stem	164.30 ± 133.77	20.17 ± 5.13	156.00 ± 22.23	1.15 ± 0.02
S11	CA:G Leaves	51.30 ± 30.29	10.26 ± 0.80	>1000	0.95 ± 0.06
S12	CA:G Stem	273.41 ± 217.76	30.00 ± 11.91	137.74 ± 57.46	1.24 ± 0.03
S13	CA:F Leaves	163.14 ± 107.12	6.04 ± 0.82 *	268.27 ± 161.03	0.92 ± 0.04
S14	CA:F Stem	374.70 ± 130.74	13.54 ± 4.34	131.77 ± 70.93	2.97 ± 0.02
S15	CA:S Leaves	587.96 ± 302.14	12.14 ± 7.54	554.85 ± 362.96	0.95 ± 0.05
S16	CA:S stem	>1000	54.46 ± 28.12	-	-
S17	MA:G Leaves	>1000	8.79 ± 2.17 *	-	-
S18	MA:G Stem	>1000	34.03 ± 15.08	-	-
S19	MA:F Leaves	>1000	6.73 ± 1.48 *	-	-
S20	MA:F Stem	>1000	27.48 ± 0.59	-	-
S21	MA:S Leaves	>1000	18.56 ± 3.18	-	-
S22	MA:S Stem	>1000	39.47 ± 20.13	-	-
	Ascorbic acid	0.08 ± 0.03	0.08 ± 0.06	1.77 ± 0.15	0.034 ± 0.003

* = difference of IC_50_ value on free radical scavenging and lipid peroxidation inhibition with significant difference at *p* < 0.05 between S1 and DESs extracts.

**Table 3:** Metal chelating activity, ABTS scavenging activity, and ferric reducing antioxidant power (FRAP) of DES extracts.

Code	Components	%Metal Chelating Activity	%ABTS Scavenging Activity	FRAP Value
S1	Water Leaves	59.42 ± 3.38	18.79 ± 1.18	251.02 ± 11.38
S2	Water Stem	45.08 ± 6.95	-	-
S3	95% EtOH Leaves	54.78 ± 5.28	-	-
S4	95% EtOH Stem	60.97 ± 2.70	-	-
S5	50% EtOH Leaves	33.81 ± 0.87	-	-
S6	50% EtOH Stem	38.40 ± 3.58	-	-
S7	LA:G Leaves	24.86 ± 1.31	20.72 ± 1.43	139.24 ± 2.83
S8	LA:G Stem	21.97 ± 6.62	10.41 ± 058	3.67 ± 1.80
S9	LA:F Leaves	29.23 ± 1.24	24.91 ± 2.47	129.83 ± 9.00
S10	LA:F Stem	25.46 ± 5.63	12.90 ± 0.56	31.69 ± 7.50
S11	CA:G Leaves	12.95 ± 1.94	14.65 ± 0.13	-
S12	CA:G Stem	15.57 ± 0.51	5.08 ± 0.50	-
S13	CA:F Leaves	19.72 ± 0.01	14.30 ± 1.03	-
S14	CA:F Stem	20.42 ± 1.47	8.29 ± 0.40	-
S15	CA:S Leaves	18.37 ± 1.72	8.86 ± 0.25	-
S16	CA:S stem	14.80 ± 5.95	-	-
S17	MA:G Leaves	8.18 ± 1.78	-	-
S18	MA:G Stem	9.63 ± 1.48	-	-
S19	MA:F Leaves	11.13 ± 1.45	-	-
S20	MA:F Stem	7.88 ± 2.43	-	-
S21	MA:S Leaves	10.66 ± 2.45	-	-
S22	MA:S Stem	3.97 ± 1.16	-	-
	Ascorbic acid	-	99.69 ± 0.24	2358.77 ± 8.45
	EDTA	99.83 ± 0.03	-	-

**Table 4:** Percentage of cell viability and IC_50_ values after exposure to selected DESs extracts.

Code	Concentration (mg/mL)	% Cell Viability	IC_50_ (mg/mL)
S1 Water Leaves	0.27	91.96 ± 4.89	127.81 ± 41.43
	0.55	90.53 ± 2.65	
	1.1	89.58 ± 5.06	
	2.2	83.26 ± 4.30	
	4.39	76.09 ± 2.31	
S7 LA:G Leaves	0.25	97.64 ± 5.47	9.20 ± 0.15
	0.51	95.50 ± 6.24	
	1.02	101.46 ± 4.80	
	2.03	98.82 ± 4.15	
	4.06	89.20 ± 2.56	
S9 LA:F Leaves	0.23	96.74 ± 5.43	8.00 ± 0.19
	0.46	94.83 ± 5.11	
	0.93	92.14 ± 1.33	
	1.85	92.69 ± 4.51	
	3.71	79.77 ± 7.42	
S11 CA:G Leaves	0.25	90.06 ± 7.74	8.28 ± 0.45
	0.49	95.74 ± 1.48	
	0.98	91.45 ± 6.62	
	1.97	92.74 ± 1.70	
	3.93	76.28 ± 4.72	
S13 CA:F Leaves	0.26	95.55 ± 9.72	8.80 ± 1.48
	0.52	99.66 ± 4.81	
	1.04	96.23 ± 5.16	
	2.07	93.53 ± 9.67	
	4.14	87.84 ± 9.99	

**Table 5:** Rutin and kaempferol content in selected DESs extracts determined by HPLC assay.

Samples	Rutin Content (mg/g Extract)	Kaempferol Content (mg/g Extract)
S1 Water Leaves	8.95 ± 0.03	-
S7 LA:G Leaves	7.87 ± 0.01	25.36 ± 0.08
S13 CA:F Leaves	6.39 ± 0.01	24.78 ± 0.10

**Table 6:** Description of eight formulations generated according to 2^3^ full factorial design and response variables value from factorial design.

Formulations	Factor Variables			Response Variables			
	DES Extract	Emulsifier	Emollient	Viscosity (Pa.s)	Spreadability (g)	Cohesiveness (g)	Consistency (g.s)
F1	1	2	30	1.48 ± 0.13	18.27 ± 0.25	10.67 ± 0.14	45.48 ± 2.93
F2	1	5	30	5.15 ± 0.38	50.25 ± 4.12	25.17 ± 1.76	125.51 ± 14.56
F3	1	2	15	2.87± 0.30	23.50 ± 1.65	11.93 ± 0.68	58.05 ± 0.14
F4	5	2	15	3.29 ± 0.17	21.68± 1.17	10.23 ± 0.12	55.55 ± 6.10
F5	5	5	30	5.40 ± 0.26	40.02 ± 0.81	18.87 ± 1.06	96.31 ± 4.97
F6	5	5	15	5.30 ± 0.39	44.08 ± 1.04	17.54 ± 1.40	103.77± 0.52
F7	1	5	15	4.34 ± 0.28	32.80 ± 0.81	13.44 ± 0.14	76.28 ± 2.21
F8	5	2	30	1.59 ± 0.07	15.50 ± 0.51	9.38± 0.32	40.63 ± 1.47

**Table 7:** Estimated regression coefficient of optimized formulation.

Responses	Viscosity	Spreadability	Cohesiveness	Consistency
**Linear terms**				
Intercept (β_0_)	3.68	30.89	14.65	75.93
β_A_	0.2175	-	−0.6488	-
β_B_	1.37 *	10.90 *	4.10 *	24.54 *
β_C_	−0.2739 *	-	1.37 *	-
**Interaction terms**				
β_AB_	-	-	-	-
β_AC_	-	−2.68	−1.25	−6.65
β_BC_	0.5005	3.2	1.90 *	9.38
β_ABC_	-	-	−1.35 *	-
**Model**				
F-value	64.06	21.88	431.34	15.51
*p*-value	0.0031	0.0061	0.0368	0.0114
R^2^	0.9884	0.9462	0.9996	0.9209
Adjusted R^2^	0.9730	0.8995	0.9973	0.8615
Predicted R^2^	0.9177	0.7703	0.9753	0.6834
Adequate Precision	19.9317	11.6591	59.4407	10.2135

* = significant terms at *p* < 0.05. β_A_, β_B_, and β_C_ = effect of DES extract, emulsifier, and emollient, respectively. β_AB_, β_AC_, β_BC_, and β_ABC_ = interaction between DES extract, emulsifier, and emollient that affects response variables.

**Table 8:** Comparisons of control formulation and optimized formulation (F3) of response variables under optimized conditions.

Ingredients	Total 100 g		
Hydrogenated lecithin *(B)*	3	2	
Caprylic/capric triglycerides *(C)*	25	15	
DES extract *(A)*	0	1	
Xanthan gum	1	1	
Glycerin	10	10	
Phenoxyethanol	1	1	
DI Water	60	70	
**Responses**	**Control formulation**	**Optimized formulation (F3)**	**%RSE**
Viscosity (Pa.s)	2.74 ± 0.12	2.87 ± 0.30	4.74
Spreadability (g)	21.31 ± 0.60	23.50 ± 1.65	10.28
Cohesiveness (g)	12.10 ± 0.31	11.93 ± 0.68	1.40
Consistency (g.sec)	54.43 ± 2.70	58.05 ± 0.14	6.65

**Table 9:** Formulation characterizations before and after heating–cooling cycle test.

Characterizations	Control Formulation		F3	
	Before	After	Before	After
Viscosity (Pa·s)	2.74 ± 0.12	1.91 ± 0.10	2.87 ± 0.30	2.15 ± 0.10
Spreadability (g)	21.31 ± 0.60	19.56 ± 0.81	23.50 ± 1.65	16.85 ± 0.49
Cohesiveness (g)	12.10 ± 0.31	9.70 ± 0.31	11.93 ± 0.68	7.22 ± 0.19
Consistency (g·s)	54.43 ± 2.70	48.26 ± 1.35	58.05 ± 0.14	42.95 ± 1.43

**Table 10:** Abbreviation, molar ratio of components, and appearance of DESs.

Abbreviation	Components	Molar Ratio	Appearance
LA:F	Lactic acid (88%):Fructose	5:1	Light clear yellow liquid
LA:G	Lactic acid (88%):Glucose	5:1	Transparent liquid
CA:S	Citric acid:Sucrose	1:1	Transparent liquid
CA:F	Citric acid:Fructose	1:1	Light clear yellow liquid
CA:G	Citric acid:Glucose	1:1	Transparent liquid
MA:G	Malic acid:Glucose	1:1	Transparent liquid
MA:F	Malic acid:Fructose	1:1	Light clear yellow liquid
MA:S	Malic acid:Sucrose	1:1	Light clear yellow liquid

**Table 11:** Composition of control emulsion formulation.

Ingredients	Function	Total 100 g
**Oil phase**		
Hydrogenated lecithin	Emulsifier	3
Caprylic/capric triglycerides	Emollient	25
**Water phase**		
DES extract	Active	0
Xanthan gum	Thickener	1
Glycerin	Humectant	10
Phenoxyethanol	Preservative	1
DI Water	Solvent	60

**Table 12:** Experimental runs for formulations used in study with coded values.

Independent Variables	Name	Unit	Level	
			Low (−)	High (+)
A	DES extract	%, *w*/*w*	1	5
B	Emulsifier	%, *w*/*w*	2	5
C	Emollient	%, *w*/*w*	15	30

## Data Availability

The authors confirm that the data are contained within the article.
